# Predicting the Performance Deterioration of a Three-Shaft Industrial Gas Turbine

**DOI:** 10.3390/e24081052

**Published:** 2022-07-31

**Authors:** Waleligne Molla Salilew, Zainal Ambri Abdul Karim, Tamiru Alemu Lemma, Amare Desalegn Fentaye, Konstantinos G. Kyprianidis

**Affiliations:** 1 Mechanical Engineering Department, Universiti Teknologi PETRONAS, Bandar Seri Iskandar 32610, Perak, Malaysia; waleligne_20001048@utp.edu.my; 2 Centre for Automotive Research and Electric Mobility (CAREM), Universiti Teknologi PETRONAS, Seri Iskandar 32610, Perak, Malaysia; ambri@utp.edu.my; 3 School of Business, Society and Engineering, Mälardalen University, 883, SE-72123 Vasteras, Sweden; amare.desalegn.fentaye@mdu.se (A.D.F.); konstantinos.kyprianidis@mdh.se (K.G.K.)

**Keywords:** gas turbine, design point, off-design, steady-state, performance, physical faults

## Abstract

The gas turbine was one of the most important technological developments of the early 20th century, and it has had a significant impact on our lives. Although some researchers have worked on predicting the performance of three-shaft gas turbines, the effects of the deteriorated components on other primary components and of the physical faults on the component measurement parameters when considering the variable inlet guide valve scheduling and secondary air system for three-shaft gas turbine engines have remained unexplored. In this paper, design point and off-design performance models for a three-shaft gas turbine were developed and validated using the GasTurb 13 commercial software. Since the input data were limited, some engineering judgment and optimization processes were applied. Later, the developed models were validated using the engine manufacturer’s data. Right after the validation, using the component health parameters, the physical faults were implanted into the non-linear steady-state model to investigate the performance of the gas turbine during deterioration conditions. The effects of common faults, namely fouling and erosion in primary components of the case study engine, were simulated during full-load operation. The fault simulation results demonstrated that as the severity of the fault increases, the component performance parameters and measurement parameters deviated linearly from the clean state. Furthermore, the sensitivity of the measurement parameters to the fault location and type were discussed, and as a result they can be used to determine the location and kind of fault during the development of a diagnosis model.

## 1. Introduction

The gas turbine (GT) is one of the most expensive types of equipment in aviation and stationary mechanical applications, and it is widely used in the oil, gas, petrochemical, and power generation industries, where reliability and accessibility are two of the most important attributes [1,2]. It is a thermal machine and a complex piece of machinery with mechanical, electrical, and hydraulic systems that operate at high speeds, temperatures, pressures, and stresses [3]. This complex equipment is the most critical innovation, and it has changed the world in numerous ways. A gas turbine’s performance degrades gradually with time due to the hostile operating conditions. To minimize the related increases in energy consumption and environmental pollution, anomalous working conditions need to be avoided as early as possible. This requires effective condition monitoring and diagnostic techniques that can detect, isolate, and assess impending or incipient failure conditions and suggest the appropriate maintenance actions at the right time. Improving a gas turbine’s operating performance will have a significant impact on its energy consumption and environmental pollution. A gas turbine’s performance degrades due to different physical faults, such as fouling, erosion, corrosion, domestic object damage, and foreign object damage [4,5]. The causes of fouling in an engine include dust, dirt, sand, rust, ash, carbon particles, chemicals, and fertilizers. This fouling decreases the compressor and turbine flow capacity, efficiency, and pressure ratio [6,7]. Erosion occurs in a gas turbine due to the presence of dirt, sand, dust, ash, and carbon particles, and it decreases the compressor flow capacity, efficiency, and pressure ratio but increases the turbine flow capacity [8,9]. Salts, acids, nitrates, and sulfates cause corrosion, which decreases the compressor flow capacity, compressor, and turbine efficiency, while the turbine flow capacity increases [10,11]. Foreign object damage (FOD) and domestic object damage (DOD) is caused due to the existence of hailstones, runway gravel or birds, large carbon particles, and internal braking in the gas turbine. The existence of such things decreases the compressor and turbine efficiency and pressure ratio, whereby the compressor and turbine flow capacity are increased or decreased [9,12]. In addition to physical faults, the gas turbine performance is deteriorated due to ambient condition variations, variable inlet guide vale drift, and bleed air leakage [13].

To evaluate the performance of a gas turbine due to a physical fault, ambient condition variations, variable inlet guide valve drift and bleed air leakage, several studies have been conducted to investigate performance deviations from the clean condition. Sepehr et al. [14] developed a new method for determining the ideal values of design parameters for industrial twin-shaft gas turbines, variable-geometry turbines, and compressors, with fuel control operations at varying ambient temperatures also being described. The focus of their research was an on–off design with varying ambient temperatures. They used the power output in the objective function optimization procedure with a genetic algorithm and applied characteristic compressor and turbine maps and the thermodynamic matching approach to model the gas turbines. Muhammad et al. [15] developed a steady-state off-design model for a single-shaft industrial gas turbine with a variable geometry to study the cumulative impact of fouling and shifting ambient conditions on the gas turbine’s performance. The researchers proved that a rise in the ambient intake air temperature causes a decrease in the performance of the gas turbines in hot and humid circumstances. Finally, the scholars concluded that the performance deterioration of the industrial gas turbines due to the ambient condition variations, especially in hot climate zones, can be improved by integrating inlet air cooling measures with variable-geometry engines. Chen et al. [16] developed a sequential model-based method for diagnosing the performance of gas turbines. The suggested method has an advantage over the previous model-based approaches that employ the same number of measurements, in that it can more accurately and efficiently assess the degree of engine component degradation, such as compressor fouling and turbine erosion. The result show that with a smaller set of measurements, the new approach provides an accurate diagnosis.

Diakunchak [8] developed the most valuable gas turbine diagnosis model. His study separated the recoverable deterioration and unrecoverable deterioration into two categories for gas turbine performance degradation. He also investigated the key causes of performance decline and the changes in performance indicators. He also provided techniques for tracking and forecasting the decline in gas turbine performance. Merrington et al. [17] used a fundamental concept to create a mathematical gas turbine performance model to relate the measured variables, and then to track how faults occur that affect the model parameters. The results obtained demonstrated a significant advancement over those achieved using conventional techniques. Escher [18] developed a gas turbine performance model and drew his suggestions for the investigation of typical gas path faults and the relationship between faults and key performance indicators. Additionally, he created the software PYTHIA and used Newton–Raphson solution techniques to build and solve a performance deterioration model for gas turbines. Additionally, the design of the gas path fault diagnosis system utilized the performance deterioration results. Saravanamuttoo and Maclsaac [19] presented a performance deterioration model, which primarily simulated performance deterioration and was built based on the standard gas turbine model, to examine the relationship between health parameters and measurement parameters. In this study, gas turbine gas path faults were simulated using a modified deterioration component characteristic curve. Kurz and Brun [20]. assessed how the gas turbine performance is affected by the industrial gas turbine compressor and turbine physical faults. Ping et al. [21] created a thermodynamics model for pipe gas turbines and investigated the impacts of faults in a gas engine and the fault detection of gas turbines. Lakshminarasimha et al. [22] created gas turbine models and investigated the impacts of erosion in an aviation gas turbine and fouling in a power plant gas turbine. MacLeod et al. [23] developed a gas turbine performance model and investigated how common physical faults affected each individual engine component’s performance. Aker et al. [24] developed a mathematical model for a gas turbine to simulate the fouling of compressor blades to collect gas path fault characteristics for fault detection and to reduce unnecessary unplanned maintenance costs and operating expenses. Additionally, he analyzed the impacts of compressor fouling on key performance indicators for gas turbines.

As stated above, researchers have investigated the simulation of gas turbine performance degradation and gas path faults, but some of them have only examined one component, while others have only studied two. However, only a small number of researchers have examined how all major gas turbine components degrade in performance. Furthermore, the previous research has only studied the performance decline of gas turbines under full-load conditions, ignoring part-load operations. Kurz and Brun [25] developed a gas turbine model for single- and two-shaft gas turbine engines. They investigated how the gas turbine performance is impacted by degradation and how it arises. The focus of the study was on the gas turbine components as a system because the function of a gas turbine is the consequence of the fine integration of all components. As a result, a detailed analysis was carried out on the component interactions. Different types of physical faults were caused to show how the component degradation affects the engine compressor’s operating points, the performance characteristics of gas turbines running at full- and part-loads, and the impacts of the component degradation on the engine measurement parameters. Finally, to provide a direction for condition monitoring, parameters that indicate the levels of degradation were established. Mohammadi et al. [26] created a simulation model for two-shaft gas turbines to solve this issue. He focused on three major components, the compressor, gas generator turbine, and power turbine, and investigated the impacts of typical gas path faults on the gas turbine performance under full and part loads. Qingcai et al. [27] developed a non-linear gas turbine model to simulate and investigate the effects of fouling and erosion on the gas turbine performance. The engine model was not mentioned in the paper, but it was mentioned that the gas turbine engine was a three-shaft gas turbine with a total pressure ratio of 11.8:1, four-stage axial flow low-pressure compressors in groups of four, seven-stage high-pressure compressors, and with an engine power output of 11.5 MW. According to the results of the fault simulation under part- and full-load conditions, the component performance parameters and the measurement parameters will vary almost linearly from the clean condition as the fault severity increases.

**Figure 1:**
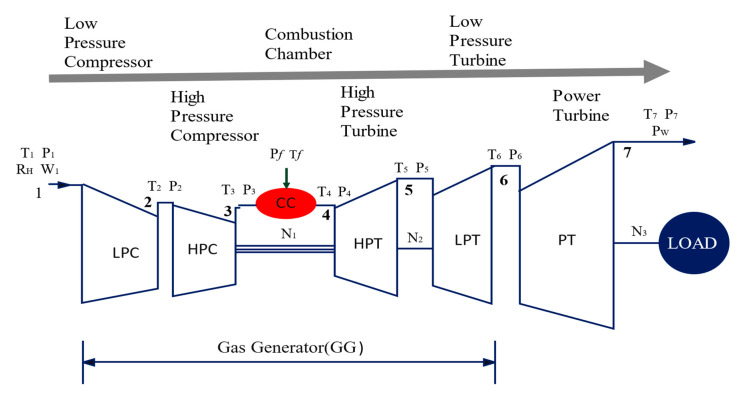
Configuration of a three-shaft gas turbine with stations.

Hence, this research aimed to investigate the effects of physical faults on the performance of a three-shaft gas turbine with variable inlet guide vane scheduling and a secondary air system or cooling system. The gas turbine design point and off-design performance model were developed and validated with the engine manufacturer’s data. Fouling and erosion faults were implanted into the clean developed model with the help of the relationship between the physical faults and performance parameters. The effects of physical faults on the output and performance parameters were investigated. Furthermore, the study investigated the effect of a faulty component on the performance of another integrated component. Finally, the measurement deviation due to physical faults was discussed and the diagnosis set parameters for each component diagnosis were pointed out. The case engine in this study is a three-shaft gas turbine engine. It has six main gas path components, including a low-pressure compressor (IPC), high-pressure compressor (HPC), combustion chamber, high-pressure turbine (HPT), low-pressure turbine (IPT), and power turbine (PT) [28]. It is equipped with a two-shaft gas generator and a single-shaft power turbine. The gas generator is equipped with low- and high-pressure compressors as well as low- and high-pressure turbines. The low-pressure axial turbine powers the low-pressure compressor, while the high-pressure turbine powers the high-pressure compressor. The power turbine is a free axial turbine with two stages. The cooling air is extracted from the mid and final stages of the low and high-pressure compressors. The three-shaft gas turbine configuration is shown in Figure 1.

## 2. Performance Development of a Gas Turbine Engine

**Figure 2:**
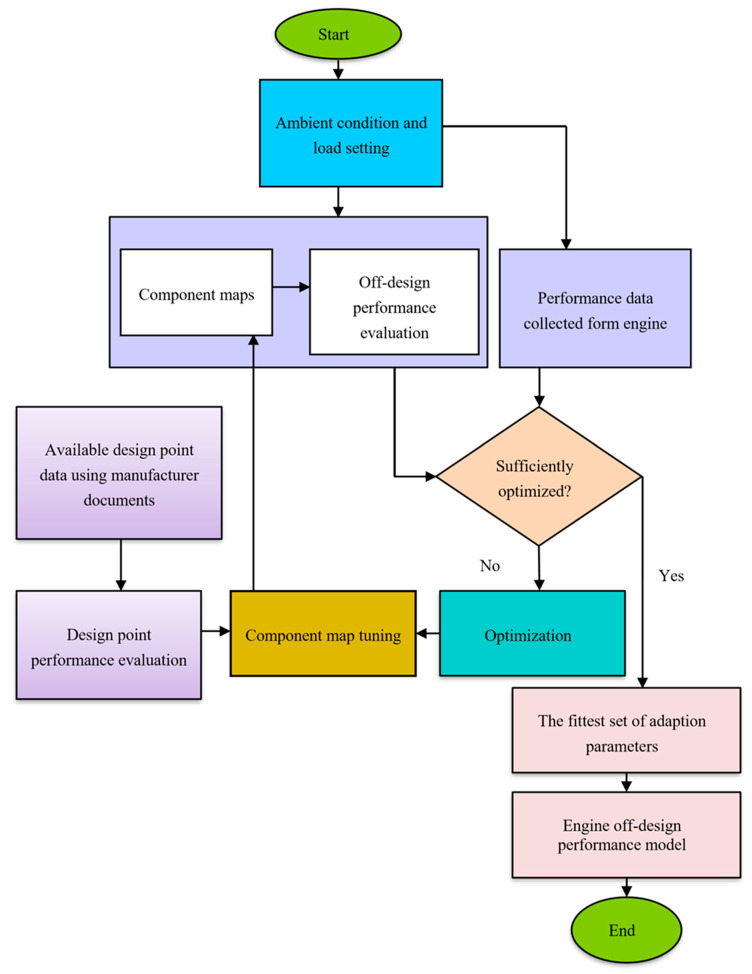
Gas turbine performance evaluation flowchart.

A gas turbine performance model needs two components—a design point model and an off-design model [13]. The design point model is a simulation of the engine at full load, calculating all parameters at each station of components. It is a single operating point and is considered one operating point in an off-design performance simulation. The design point performance of the actual gas turbine cycle is determined by combining the performance of distinct components [29,30]. A cost-effective solution to this challenge is to reduce the experimental data to a minimum and acquire the remaining values using gas turbine performance modelling [31]. The accessible data in this inquiry is confined to a few critical design point performance data points and a small amount of gathered data. As a result, the technique depicted in Figure 2 is recommended for evaluating the component load performance. These certain design point data were collected from the manufacturer’s technical manuals or catalogues. To complete the collection of the design point performance data, an energy-based design point assessment approach was applied [32]. The model is confirmed if the generated results are close to those obtained directly from the gas turbine engine [33]. Otherwise, the parameters that govern the geometry of the component maps can be optimized using an optimization technique.

### 2.1. Design Point Performance Model

**Figure 3:**
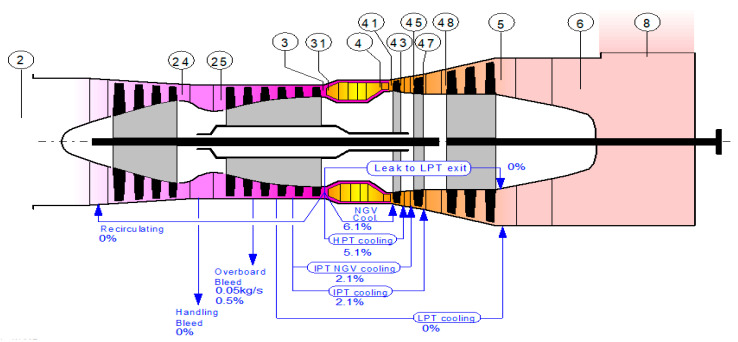
Secondary air fraction used during the simulation.

**Figure 4:**
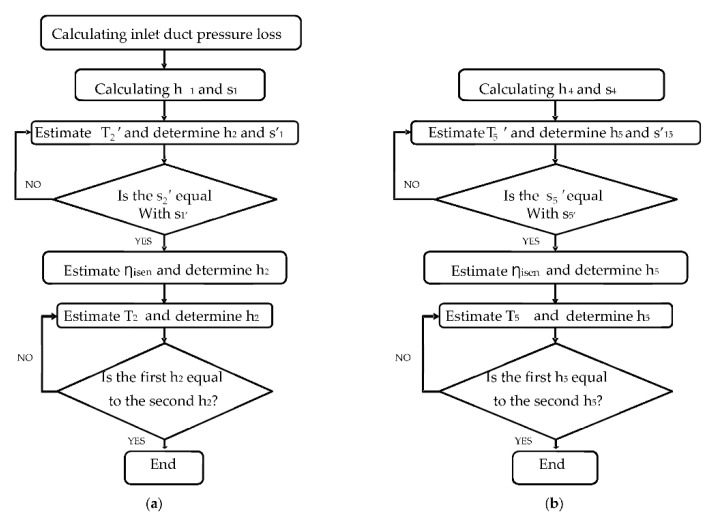
Design point performance evaluation flowchart: (**a**) low-pressure compressor; (**b**) high-pressure turbine.

The purpose of the design-point calculations for the industrial gas turbine engines was to determine the parameters for the maximum power output of the engine. The input data included the ambient conditions, compressor pressure ratio, turbine entry temperature (or air–fuel ratio in the combustion chamber), and component efficiencies. However, due to proprietary issues, engine manufacturers do not provide all of the necessary data for end-users. Thus, the design point performance model develops the engine performance model at a reference (single) operating point and by calculating unknown parameters using thermodynamic equations. The values of the secondary air system used when developing the performance model are shown in Figure 3. The compatibility or the energy balance between the components of the common shaft must be assessed. These thermodynamics equations are programmed using MATLAB or other programing languages. The model is then optimized until the percentage error becomes lower and the energy becomes balanced. The most accurate method for developing a gas turbine performance model is using the enthalpy–entropy approach. GasTurb 13 commercial software uses this accurate method. A flowchart of the deign performance modeling process for a low-pressure compressor and high-pressure turbine is shown below in Figure 4a,b. The high-pressure compressor, low-pressure turbine, and power turbine are modeled in the same fashion. The input data for the design and off-design performance modeling and validation of the model are collected from different sources. Most of the data are collected from engine manufacturer datasheets, such as the power output, spool speed, thermal efficiency inlet mass flowrate, exhaust temperature, pressure ratio, heat rate, compressor, and turbine stage data. The rest of the input parameters are collected from published papers and engineering judgements, or estimated values are used during the optimization of the model. To validate the off-design model over the entire operating range, the data collected from the engine catalogue or datasheet are used, such as the power output versus the ambient temperature and the thermal efficiency versus the ambient temperature. The ambient condition is based on the international organization for standardization (ISO) design point standards. Generally, the gas turbine parts, including the compressors, combustors, and turbines performance models, are developed and their actual thermodynamic performances are assessed separately. Then, the actual gas turbine cycle’s design point (single operating point) performance will be determined by adding the performances of the separate components. The data to be calculated in the design point simulation include the thermodynamic properties such as *P* and *T* at different gas path points, the compressor inlet air flow rate, the fuel flow rate, the components’ isentropic efficiencies, the gas generator speed, the power turbine speed, and the net power output. Some of these parameters were accessible from the manufacturer’s technical documents and catalogues. The unavailable parameters that were needed to be figured out included P2, *T*2, P2, T3, P4, *T*3, *P*4, T5, *P*5, T6, P6, P7, *ẇf*, and each component’s efficiency. All of these parameters were simulated using the input data shown in Table 1. The model was optimized using the optimization parameters and the constraints are shown in Table 2 and Table 3 respectively. Table 4 is objective function or Figure of merit.

With the known input parameters found from the catalogue and open literature, and with some engineering judgements, the design point performance was simulated with GasTurb 13 [35]. The model was optimized until it matched with the validation. Finally, it was observed that the model matched with the engine manufacturer data with quite small deviations.

The values for the overboard bleed air, HPT NGV 1 cooling, HPT rotor 1 cooling, IPT NGV 1 cooling, and IPT rotor 1 cooling were about 0.5%, 5%, 3%, 1%, and 1%, respectively. The secondary air fractions were estimated by considering other three-shaft engine design point performance models.

Where h_1_ and s_1_ are the low-pressure compressor inlet enthalpy and entropy, respectively; h’_1_ and s’_1_ are the low-pressure compressor isentropic inlet enthalpy and entropy, respectively; s’_2_ is the low-pressure compressor isentropic exit entropy; h_2_ is the low-pressure compressor isentropic exit enthalpy; T_2_ and T’_2_ are the low-pressure compressor isentropic exit temperature and isentropic exit temperature, respectively; Ƞ_isen_ is the low-pressure compressor isentropic efficiency. All these parameters will be calculated during the development of a high-pressure turbine performance model. The inlet parameter subscript will be 4 and the exhaust parameter subscript will be 5. The energy balance equation is used to develop the combustion chamber performance model, and the pressure loss is considered:

(1) $${\dot{m}}_{a}h_{3}+{\dot{m}}_{f}\times LHV\times \eta_{cc}=\left({\dot{m}}_{a}+{\dot{m}}_{f}\right)h_{4}$$

(2) $${\dot{m}}_{f}=\frac{{\dot{m}}_{a}\left(h_{4}+h_{3}\right)}{\eta_{cc}\times LHV-h_{4}}\;$$

The work compatibility between the components is as follows:

(3) $$\begin{array}{c} W_{HPC}=W_{HPT}\\ W_{LPC}=W_{LPT}\\ W_{PT}=W_{Load\;} \end{array}$$

where h_3_ is the high-pressure compressor exit enthalpy, h_4_ is the combustion chamber exit enthalpy, ${\dot{m}}_{a}$ is the inlet air mass flow rate, ${\dot{m}}_{f}$ is the fuel mass flow rate, LHV is the lower heating value, and $\eta_{cc}$ is the combustion efficiency. Here, $W_{HPC}$ is the high-pressure compressor’s work rate, $W_{HPT}$ is the high-pressure turbine’s work rate, $W_{LPC}$ is the low-pressure compressor’s work rate, $W_{lPT}$ is the high-pressure turbine’s work rate, $W_{PT}$ is the power turbines work rate, and $W_{\text{Load}}$ is the load.

The results of the design point calculation were compared to the design parameters from the gas turbine product catalogue. The percent error of each parameter deviation from the catalogue was quite minimal, as shown in Table 5. As a result, it was determined that the design point values provided by GasTurb 13 simulation are highly reliable because they match the actual design values with very low variance.

### 2.2. Off-Design Performance Model

**Figure 5:**
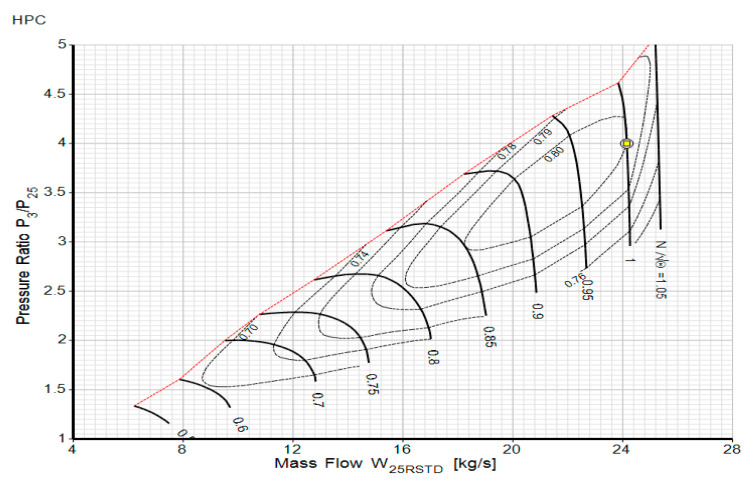
Scaled high-pressure compressor map.

The next stage was to create an off-design model using GasTurb 13 after the cycle design point calculations had been successfully approximated. The off-design performance refers to the ability of gas turbines to operate for long durations under situations other than their design settings. A change in engine load and ambient conditions might cause an off-design state. The ambient temperature, for example, may vary substantially from winter to summer, affecting the engine performance significantly. As a result, the engine must not only function well under the design settings, but it must also work well under off-design situations. Off-design simulations have two key tasks: the first task is the adaptation of the target engine’s design point with the already known compressor and turbine maps using the scaling approach; for instance, Figure 5 shows the scaling of the high-pressure compressor point to the design point. In this case, the map is scaled for the design operating point at an auxiliary beta value of 0.5 when the pressure ratio, isentropic efficiency corrected flow, and corrected speed are 4, 0.8, 24.146, and 1, respectively. All five component maps are scaled to the design point in the same fashion. The second off-design task is component matching by ensuring the mass flow and work compatibility using the Newton–Raphson iterative process [13,16]. For compressor and turbine map modifications, an appropriate characteristics map containing the design point data should be chosen. As recommended by Kurzke [32], an auxiliary coordinate will be introduced into the component map digitization procedure to eliminate disagreement. The relationship between the corrected rotational speed, corrected mass flow rate, pressure ratio, and isentropic efficiency will be shown in the component maps. The rotating speed (rpm), intake mass flow rate (kg/s), inlet temperature (K), and inlet pressure to the compressor or turbine (kPa) are represented as the main parameters in the compressor and turbine maps. To make utilizing the performance maps easier, it is normal to use the design point values to standardize the corrected speed, corrected mass flow, pressure ratio, and isentropic efficiency.

To scale the map, such as for a high-pressure compressor, the values read from the map table for the corrected mass flow, efficiency, pressure ratio, and corrected speed need to be corrected for the Reynolds number effects and Reynolds number index (RNI) with the terms fη,RNI and fW,RNI to be comparable with the design point efficiency η_dp_ and corrected flow. In GasTurb, as a default, the auxiliary point beta for the map scaling point ßR,map is equal to 0.5 and the corrected speed N√ΘR,map is equal to 1.0. The map scaling point is used as a reference point (subscript R,map), with which the design point (subscript dp) is matched:

(4) $$\eta_{\mathrm{dp},\mathrm{map}\;}=\eta_{R,map}\cdot f_{\begin{array}{c} \eta ,RNI \end{array}}\;\;\;\;\;\;\;\;\;$$

(5) $${\begin{array}{c} \left(W\sqrt{\Theta_{R}}/\delta \right) \end{array}}_{dp,\;\mathrm{map}\;}={\left(W\sqrt{\Theta_{R}}/\delta \right)}_{R,\;\mathrm{map}\;}\cdot f_{W,RNI}\;\;\;$$

where η_dp, map_ is the scaled map design point efficiency, η*_R_*,_map_ is the reference point efficiency from the unscaled map, fη,RNI is the Reynolds number index used to correct the reference point efficiency read from the map, (W√(ΘR)/δ)_dp,map_ is the scaled map-corrected mass flow, (W√(ΘR)/δ)_dp,map_ is the reference-point-corrected mass flow from the unscaled map, and fW,RNI is the Reynolds number index used to correct the reference-point-corrected mass flow read from the map. The corrected temperature (Θ) and corrected pressure (δ) are expressed below:

(6) $$\theta =\frac{T_{o}}{288.15K^{\prime }}$$

(7) $$\delta =\frac{P_{o}}{101.325\mathrm{KPa}}$$

where $T_{o}$ and $P_{o}$ are the inlet temperature and pressure, respectively. To be comparable with the design point efficiency dp, the value taken from the map needs to be corrected for Reynolds number effects using the terms fη,RNI and fW,RNI. Then, the map scaling factors will be calculated as follows by assuming fη,RNI and fW,RNI, and are usually assumed as fη,RNI = 0.99 and fW,RNI = 0.995 [36].

(8) $$\;\;\;f_{\mathrm{Mass}\;}=\frac{{\left(\frac{W\sqrt{\Theta_{R}}}{\delta }\right)}_{dp}}{{\left(\frac{W\sqrt{\Theta_{R}}}{\delta }\right)}_{R,\;\mathrm{map}\;}\cdot f_{W,RNI}}\;\;\;\;\;\;$$

(9) $$f_{Eff}=\frac{\eta_{dp}}{\eta_{R,\;\text{map}\;}\cdot f_{\begin{array}{c} \eta ,RNI \end{array}}}\;\;$$

(10) $$\;\;\;f_{P_{3}/P_{2}}=\frac{{\left(P_{3}/P_{2}\right)}_{dp}-1}{{\left(\frac{P_{3}}{P_{2}}\right)}_{R,\;\mathrm{map}\;}-1}\;\;\;\;\;\;$$

(11) $$f_{\mathrm{Speed}\;}=\frac{1}{N_{R,\;\mathrm{map}\;}}\;\;$$

where f_Mass,_ f_Eff,_ f_p2/p3,_ and F_Speed_ are the mass flowrate, efficiency, pressure ratio, and speed scaling factors, respectively. P_2_ and P_3_ are the compressor inlet and exit inlet pressures, respectively. N_R_,_map_ is the compressor speed in the map. Once all of these scaling factors are calculated, the corrected mass flow, efficiency, pressure ratio, and corrected speed need to be multiplied by the scaling factors to scale the map.

At this point, the selection of the appropriate component maps and correlation of the cycle design point for accurate off-design simulations were performed. GasTurb 13 matches the components based on the interactions of components to ensure that the flow and work rate of the connected individual components are compatible. The Newton–Raphson iterative approach is the most common method used to construct a steady-state off-design operating line because of its inherent efficiencies in providing numerical solutions and ease of application to non-linear systems [16]. 

**Figure 6:**
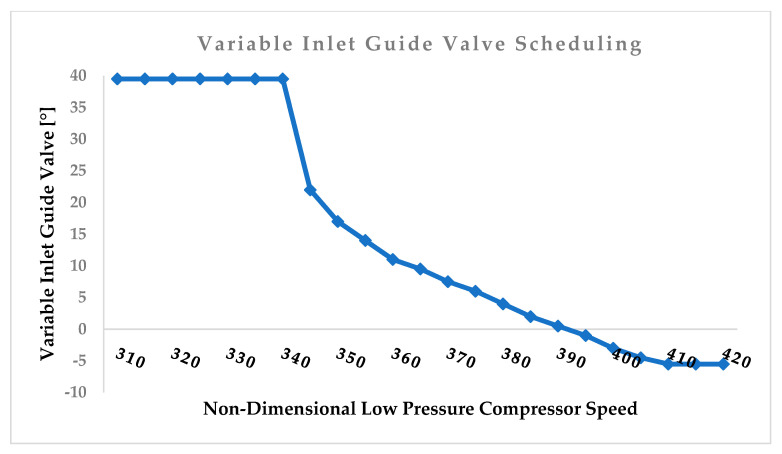
RB211−24G variable inlet guide vane scheduling.

Right after the scaling of the map to the design point, the off-design model was developed and validated with the manufacturer data. GasTurb 13, a commercial software, was used to evaluate the off-design model. The power output versus ambient temperature and efficiency versus ambient temperature from the catalogue data were used to validate the off-design model. In this research, the VIGV scheduling and bleed air were considered. The VIGV scheduling graph shown in Figure 6 was incorporated into the software during the simulation of the engine. The VIVGV scheduling data were collected from the engine manufacturer’s data. Likewise, by incorporating the VIGV scheduling data, the power output versus ambient temperature data from the engine manufacturer were also incorporated into the simulation software. This is a way of controlling the model with the power output. For the power output, it is always desired that it remains unchanged because the specific amount of power always needs to be constant throughout the operation. Even if there is a fault in the engine that deteriorates the performance, to maintain the power output the heat will be increased.

**Figure 7:**
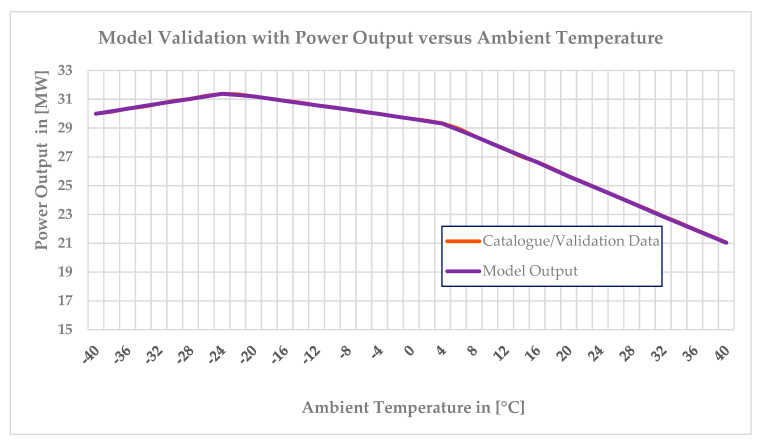
Validation of the off−design model with the power output versus ambient temperature.

**Figure 8:**
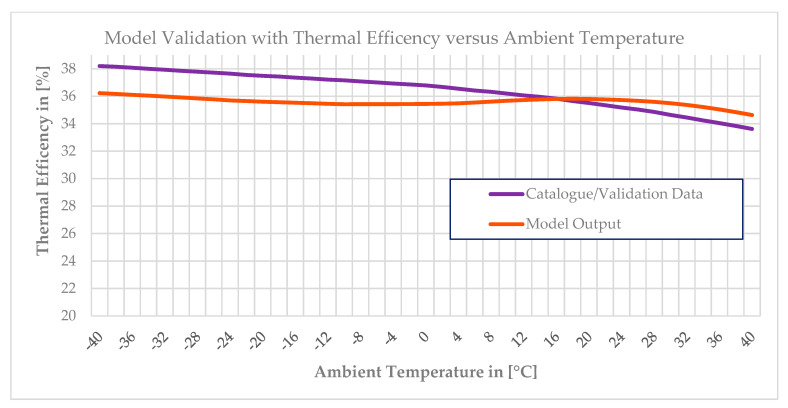
Validation of the off−design model with the thermal efficiency versus ambient temperature.

After scheduling or incorporating the above three graphs shown in Figure 5 into the software, the off-design model was used to generate data and then the generated data were compared with the validation data. Finally, the models that yielded the power output versus ambient temperature and efficiency versus ambient temperature were accurately matched with the validation data. The maximum deviation in the power output versus ambient temperature at each operating point was 0.02%, whereas a 4.5% deviation was noted in the efficiency versus ambient temperature. Comparisons between the model outputs and validation data are depicted in Figure 7 and Figure 8 below. The model output with the power output versus ambient temperature catalogue data is depicted in Figure 7 and the output with the thermal efficiency versus ambient temperature catalogue data is depicted in Figure 8 below.

Figure 7 and Figure 8 depict the off-design model results, which strongly match with the validation data. This means the model is reliable and accurate in predicting the performance of the gas turbine. There was also another option to optimize the off-design model by directly scheduling the validation data, such as the power output versus ambient temperature and efficiency versus ambient temperature, into GasTurb 13. In both approaches, the exhaust temperature versus ambient temperature and both the power output and efficiency graph are exactly similar to the first approach. The limitation of the second approach is the impossibility of simulating of the gas turbine’s performance during part-load operation. Since GasTurb 13 software is already scheduled with a power output, the limiting value option for the power output will not be active in the GasTurb software settings. If the physical faults are implanted by manipulating the flow capacity and isentropic efficiency at the power output versus ambient temperature scheduling, the output flow capacity and isentropic efficiency values will be reduced much more than the amount reduced when simulating the physical faults. To maintain the power output, more heat will be supplied, which causes an efficiency reduction. For example, to simulate fouling at 100% fault severity, the flow capacity’s isentropic and isentropic efficiency levels have to be reduced by −7.5% and −2.5%, respectively. After the fault factor is implanted, the gas generator needs to supply more heat to produce more high-temperature gas to offset the power loss caused by the decline in the component characteristics and so the output power is unchanged. Thus, the original flow capacity reduction, which was reduced by −7.5%, and the isentropic efficiency of −2.5% will not be compared with the flow or the isentropic efficiency in the normal state, but will be relative to the decreased value after the new power balance point. In short, the performance analysis is carried out by using the dynamic model. The change in component performance includes two parts: one is due to the faults, such as decreases in the flow capacity value of −7.5% and isentropic efficiency of −2.5%, and the other part is due to supplying more heat to keep the power output unchanged.

## 3. Physical Fault Simulation

The performance of the gas path components, particularly the compressor and turbine, is critical to the overall performance of the engine, and these components are important due to their vulnerability to various internal and external degradation factors. There are two reasons for the degradation of a gas turbine. The major reason might be mechanical in nature, such as misalignments, imbalances, loose components, bearing issues, or a lack of oil for lubrication. Performance-related difficulties such as fouling and debris in compressors, corrosion, edge erosion, incorrect combustion, increased clearance around the blade tip, local or domestic object damage (DOD), and thermal distortion are the second causes of gas turbine degradation. There are two types of gas turbine performance degradation, namely temporary and permanent degradation. Temporary degradation can be recovered in part during operation and an engine overhaul, whilst permanent degradation will mean the engine to be replaced. Temporary deterioration is caused by fouling, erosion, corrosion, and blade tip clearance issues, but permanent deterioration is caused by airfoil distortion and untwisting, as well as base distortions. The degradation can also be classed as recoverable (washable), non-recoverable (not recoverable with washing but can be recovered after overhaul), or permanent (neither washable nor overhaulable). The performance degradation may be categorized as long-term or short-term deterioration based on the engine’s service length or the development time frame of the deterioration.

In this paper, physical fouling and erosion faults were simulated as the most common reasons for gas turbine performance degradation. Fouling is one of the most prevalent causes of component degradation accounting for more than 70% of the total engine performance loss throughout the course of operation [23,37]. As is well known, a substantial amount of air containing particle pollutants enters the engine during gas turbine operation. The deterioration of the gas turbine engine performance is detected using health parameters. The term health parameter is used to describe the performance variations caused by various defects. The health parameter of a component is the ratio between the isentropic efficiency and mass flow during degradation and the same parameters in good conditions. The expression may be found in Equations (1) and (2). According to the calculations, the gas turbine performance degrades when the health parameter is less than 1, but not when the health parameter is more than 1 [26,27]. $H_{\Gamma ,i}$, $H_{\eta ,i}$. are the health parameters, called the flow capacity and isentropic efficiency. The physical faults were simulated with the deliberate deviation of these health parameters:

(12) $$\begin{array}{c} H_{\Gamma ,i}=\frac{{\dot{m}}_{i}\sqrt{T_{i}}}{P_{i}}/{\left(\frac{{\dot{m}}_{i}\sqrt{T_{i}}}{P_{i}}\right)}_{ref} \end{array}$$

(13) $$H_{\eta ,i}=\eta_{i}/\eta_{i}ref$$

where $H_{\Gamma ,i}$**.** **,** $H_{\eta ,i}$**.** are health parameters, called the flow capacity and isentropic efficiency, respectively; mi is the flow rate, Ti is the inlet temperature, Pi is the total pressure, and $\eta_{i}$. is the component’s isentropic efficiency.

Physical faults were implanted into the model to investigate their effects on the gas turbine performance. The relationship between the physical faults and performance parameter deviations shown in Table 6 was used. A deliberate deviation of the flow capacity and isentropic efficiency, with scheduling of the deviated values in the model as per physical faults and performance parameter relationships, was the approach used to simulate the physical faults. The deviated values were scheduled in the software using a modifier option before simulating and generating the data from the model. To make the model more concise, as stated in the Table 6, when simulating compressor fouling from 0% to 100% of the fouling severity level, the mass flow must be deliberately reduced at intervals of 0% to −7.5% and the isentropic efficiency will also be reduced at intervals of 0% to −2.5%. The variation relationship between the flow capacity and isentropic efficiency is close to 3:1 [27]. This relationship must be maintained throughout the fault severity simulation. For example, for a 10% low- and high-pressure compressor fouling severity level simulation, the flow capacity and efficiency will be decreased by −0.75% and −0.25%, respectively; that is, 10% values of −7.5% and −2.5%. For the other faults examined in this paper, each −0.2% decrease in efficiency and −0.4% in mass flow is equal to a 10% increase in fault severity. It is worth mentioning that erosion in the turbine causes an increase in flow capacity. Using this relationship, the effects of the fouling and erosion severity on the thermal efficiency, specific fuel consumption, overall pressure ratio, and exhaust temperature are simulated. The severity of the fouling is assessed for the gas generator components. Figure 6 shows the relationship between the physical faults and health parameters [26,27].

## 4. Results and Discussion

### Effects of the Fouling and Erosion on the Gas Turbine Output Parameters

Using the correlation of the physical faults and health parameters depicted in Table 6, the fouling and erosion were simulated. Using the modifier option in GasTurb 13, the physical faults were simulated. The isentropic efficiency and flow capacity are among the independent variables and changes in these independent parameters cause changes in the component performance. These performance parameters are used to quantify physical faults in the gas turbine. The dependent parameters, such as the temperature, pressure, power output, and fuel flow, then deviate as a result of the changes in the independent parameters. To quantify the fouling and erosion phenomena in the compressor and turbine, changes in the compressor’s isentropic efficiency and flow capacity (independent parameters) were studied. The component characteristics maps must be updated to account for deterioration when simulating the deterioration model. The maps already loaded in GasTurb 13, on the other hand, are for a clean engine. As a result, for a deteriorated engine, the compressor map’s scaling factors must be changed in response to changes in the independent parameters. The mass flow, isentropic efficiency, and pressure ratio scaling factors that will be calculated are listed below [27].

(14) $$F_{\Gamma ,C}=1+\frac{\Delta H_{\Gamma ,C}}{100}\;\;\;$$

(15) $$SF_{\Gamma ,C}=1+\frac{\Delta H_{\eta ,C}}{100}$$

(16) $$SF_{\Gamma ,T}=1+\frac{\Delta H_{\Gamma ,T}}{100}$$

(17) $$SF_{\eta ,T}=1+\frac{\Delta H_{\eta ,T}}{100}\;\;\;\;\;\;$$

where $SF_{\Gamma ,C}$. and $SF_{\Gamma ,C}$. describe the scaling factors used to scale the compressor flow capacity and efficiency, respectively; $SF_{\Gamma ,T}\;\mathrm{and}\;SF_{\eta ,T}$. describe the scaling factors used to scale the turbine compressor flow capacity and efficiency, respectively; $\Delta H_{\Gamma ,T}$. and $\Delta H_{\eta ,T}$. represent the variation amounts of turbine health parameters, while $\Delta H_{\Gamma ,C}$ and $\Delta H_{\eta ,C}$ are the variation quantities of compressor health parameters. After implanting physical faults using the relations, the effects of the fouling and erosion severity on the exhaust temperature, specific fuel consumption, thermal efficiency, overall pressure ratio, and turbine inlet temperature were simulated. The severity of fouling was assessed for each of the five components.

**Figure 9:**
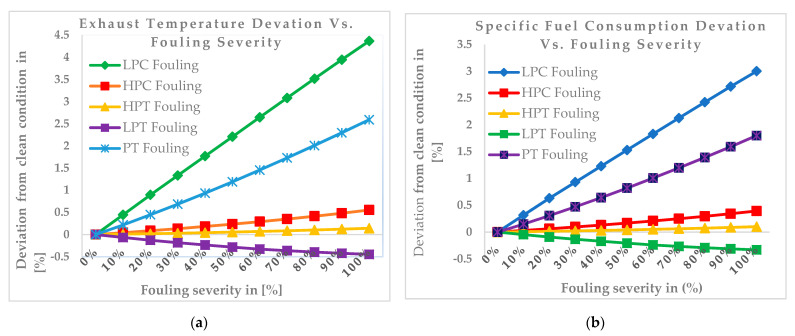

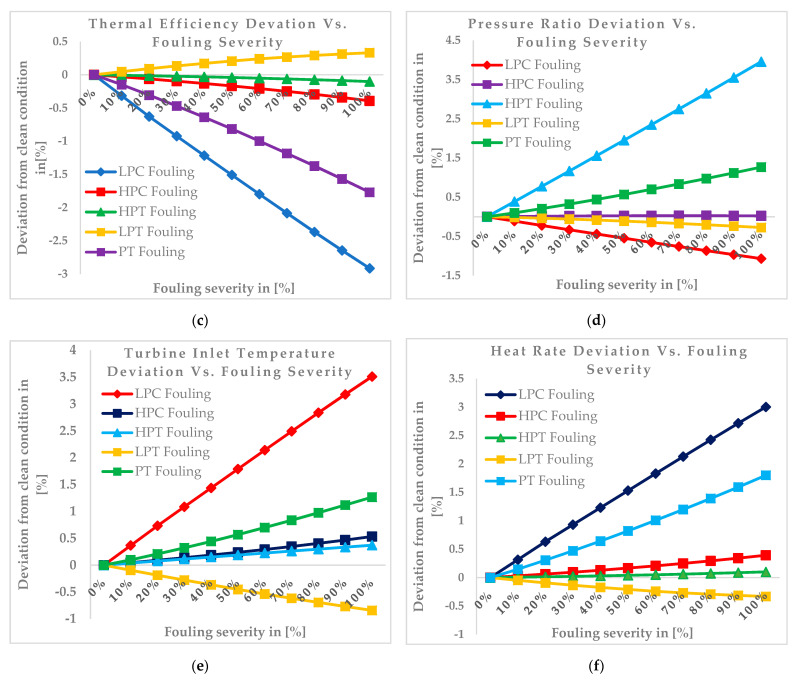
The effects of fouling on the (**a**) exhaust temperature, (**b**) specific fuel consumption, (**c**) thermal efficiency, (**d**) pressure ratio, (**e**) turbine inlet temperature, and (**f**) heat rate.

All of the numbers above clearly indicate the effects of the fouling on the gas turbine output parameters. The effect of the fouling on the exhaust temperature is depicted in Figure 9a. The maximum increase in the exhaust temperature due to fouling occurs in the low-pressure turbine, by about 4.3%, whereas it drops when the fouling occurs in the low-pressure turbine, by about −0.5%. Figure 9b depicts the effect of fouling on the specific fuel consumption rate. The deviation trend is similar to the graph of the exhaust temperature deviation due to fouling. The maximum drop in the specific fuel consumption rate due to fouling occurs in the low-pressure turbine, by about −0.5%, whereas the maximum rise occurs when the fouling occurs in the low-pressure compressor, by about 3%. Figure 9c shows the influence of the fouling on the thermal efficiency, and the result shows that the fouling decreases the thermal efficiency when it occurs in each component except the low-pressure turbine. The maximum efficiency drop occurs when fouling is implanted in the low-pressure compressor, by about −3%, where the maximum rise can be observed for the low-pressure turbine, by about 0.3%. Figure 9d depicts the effects of fouling on the overall pressure ratio. The overall pressure ratio decreases substantially when the fouling occurs in the low-pressure compressor, by about −1% at the 100% fouling severity level, whereas it rises when the fouling occurs in the high-pressure turbine, by about 4% at the 100% fouling severity level. Figure 9e depicts the effect of the fouling on the turbine inlet temperature. The deviation trend is similar to the graphs of the exhaust temperature and specific fuel consumption deviation. The maximum drop in the turbine inlet temperature due to the fouling occurs in the low-pressure turbine, by about −0.8% at the 100% fouling severity level, whereas the maximum rise occurs when the fouling occurs in the low-pressure compressor, by about 3.7% at the 100% fouling severity level. Figure 9f depicts the effect of the fouling on the heat rate. The deviation trend is similar to the graphs of the exhaust temperature, specific fuel consumption, and turbine inlet temperature deviation. The maximum drop in the heat rate due to the fouling occurs in the low-pressure turbine, by about −0.4%% at the 100% fouling severity level, whereas the maximum rise occurs when the fouling occurs in the low-pressure compressor, by about 3.1% at the 100% fouling severity level.

**Figure 10:**
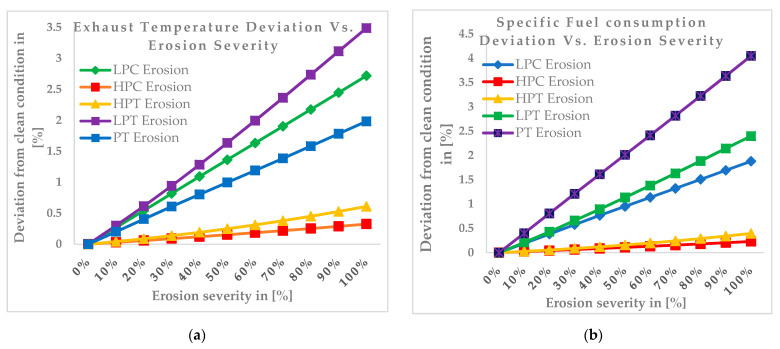

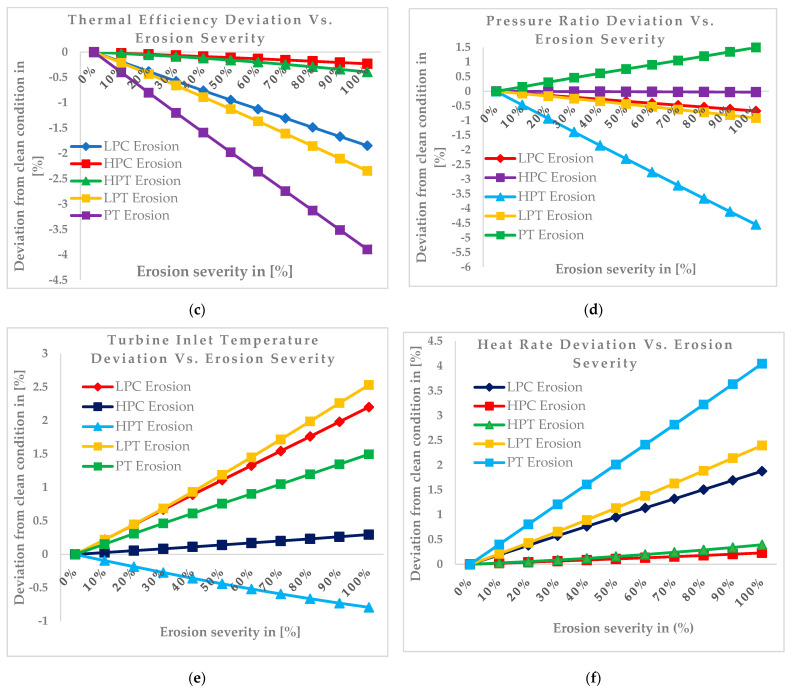
The effects of erosion on the (**a**) exhaust temperature, (**b**) specific fuel consumption, (**c**) thermal efficiency, (**d**) pressure ratio, (**e**) turbine inlet temperature, and (**f**) heat rate.

Figure 10a illustrates the effects of erosion on the exhaust temperature. As the figure shows, the maximum rise in exhaust temperature occurs when the erosion is implanted into the low-pressure turbine, by about 3.5% at the 100% erosion severity level. The minimum deviation in exhaust temperature is shown when erosion occurs in the high-pressure compressor, by about 0.3% at the 100% erosion severity level. It can be observed that the exhaust temperature rises when erosion occurs in all of the main components. The effects of the erosion on the specific fuel consumption are depicted in Figure 10b. The figure illustrates that the specific fuel consumption rises when the erosion occurs for all five components. The maximum rise in the specific fuel consumption can be observed when the erosion occurs in the power turbine, by about 4.1% at the 100% erosion severity level, whereas the minimum specific fuel consumption rise is shown when the erosion occurs in the high-pressure compressor, by about 0.2% at the 100% erosion severity level. Figure 10c illustrates the influence of the erosion on the thermal efficiency. From the results, it can be observed that a thermal efficiency decrement occurs for each component when erosion occurs. The maximum efficiency drop occurs when the erosion is implanted into the power turbine, by about −4% at the 100% erosion severity level, whereas the minimum drop can be observed for the high-pressure compressor, by about −0.2% at the 100% erosion severity level. Figure 10d depicts the effects of erosion on the overall pressure ratio. The maximum overall pressure ratio drop can be observed when the erosion occurs in the high-pressure turbine, by about −4.5% at the 100% erosion severity level, whereas it rises when the erosion develops in the pressure turbine, by about 1.5% at the 100% erosion severity level. Figure 10e depicts the effects of the erosion on the turbine inlet temperature. When the erosion occurs in the low-pressure turbine, the turbine inlet temperature increase will be higher than in other the components, but it will drop in the high-pressure turbine. The result clearly shows that the maximum turbine inlet temperature rise occurs when erosion occurs in the low-pressure turbine, by about 2.7% at the 100% erosion severity level, whereas the maximum drop can be observed when erosion occurs in the high-pressure turbine, by about −0.7% at the 100% erosion severity level. Figure 10f shows the effect of the erosion on the heat rate. As the figure shows, the maximum rise in the heat rate occurs when the erosion is implanted into the pressure turbine, by about 4% at the 100% erosion severity level. The minimum rise in the heat rate is shown when the erosion is implanted into the high-pressure compressor, by about 0.2% at the 100% erosion severity level. After assessing the effects of the fouling and erosion on the output parameters of the gas turbine engine, the effects of the fouling and erosion on component efficiency were simulated. The aim of the simulation was to predict the effects of fouling and erosion in one component on another clean component. The simulation results are depicted in the following graphs.

**Figure 11:**
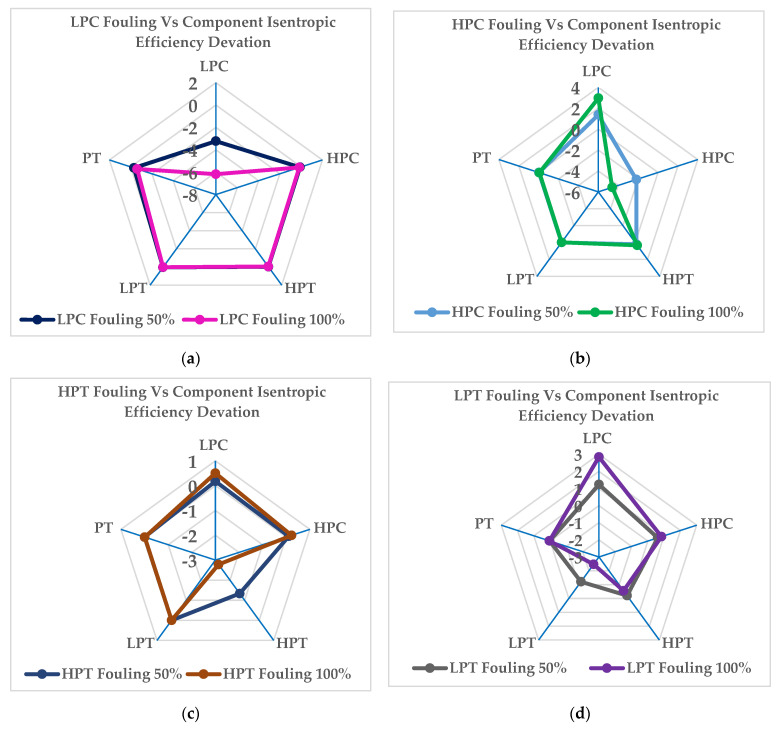

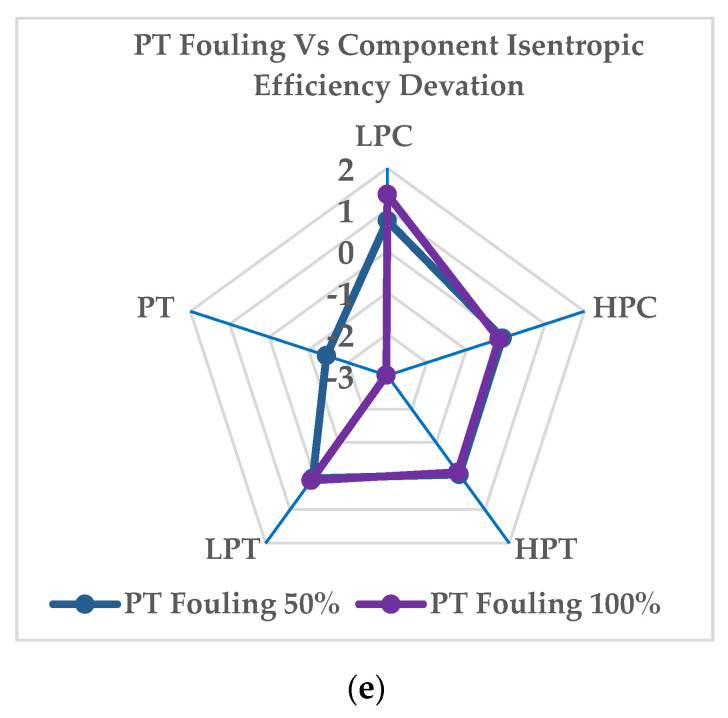
Deviation in the isentropic efficiency rates at fouling severity levels of 50% and 100% in the full−load condition for different offending components: (**a**) LPC; (**b**) HPC; (**c**) HPT; (**d**) LPT; (**e**) PT.

Figure 11a illustrates the variation in the component isentropic efficiency rates when fouling occurs in the low-pressure compressor at the fouling fault severity levels of 50% and 100%. In this simulation the maximum decreases in isentropic efficiency occur on the compressor itself, by about −3.23% at the 50% and −6.18% at the 100% severity level. The maximum effects on the deviation of the other component’s isentropic efficiency can be observed for the low-pressure isentropic efficiency at the 50% and in the power turbine at the 100% severity level, by about 0.4% and −0.6%, respectively. Figure 11b illustrates the variation in the component isentropic efficiency rates when the fouling occurs in the high-pressure compressor at fouling fault severity rates of 50% and 100%. The maximum decreases in isentropic efficiency occur on the compressor itself, by about −2.18% at 50% and −4.61% at 100%. The maximum deviation in the other component’s isentropic efficiency can be observed in the low-pressure compressor, by about 1.39% at 50% and about 2.96% at 100%. The lowest effects can be observed in the low-pressure turbine, by about −0.0083% at 50% and about −0.022% at the 100% severity level. Figure 11c illustrates the variation in the component isentropic efficiency when fouling occurs in the high-pressure turbine at fouling fault severity levels of 50% and 100%. The maximum decreases in isentropic efficiency can be observed in the high-pressure turbine, by about −1.33% at 50% and about −2.78% at the 100% severity level. The maximum deviation of the other component’s isentropic efficiency was observed in the low-pressure compressor, by about 0.17% at 50% and about 0.5% at the 100% severity level. Figure 11d illustrates the variation in component isentropic efficiency rates when fouling occurs in the low-pressure turbine at fouling fault severity levels of 50% and 100%. The maximum decreases in isentropic efficiency occur in the low-pressure turbine, by about −1.21% at 50% and about −2.46% at the 100% severity level. The effect on the deviation of the other component’s isentropic efficiency is maximal in the low-pressure power compressor, by about 1.23% at 50% and about 2.84% at the 100% severity level. Figure 11e illustrates the variation in the component’s isentropic efficiency when fouling occurs in the pressure turbine at fouling fault severity levels of 50% and 100%. The maximum decreases in isentropic efficiency are shown in the power turbine, by about −1.45% at 50% and about −2.97% at the 100% severity level. The effect on the deviation of the other component’s isentropic efficiency is maximal in the low-pressure compressor, by about 0.73% at 50% and about 1.36% at the 100% severity level.

**Figure 12:**
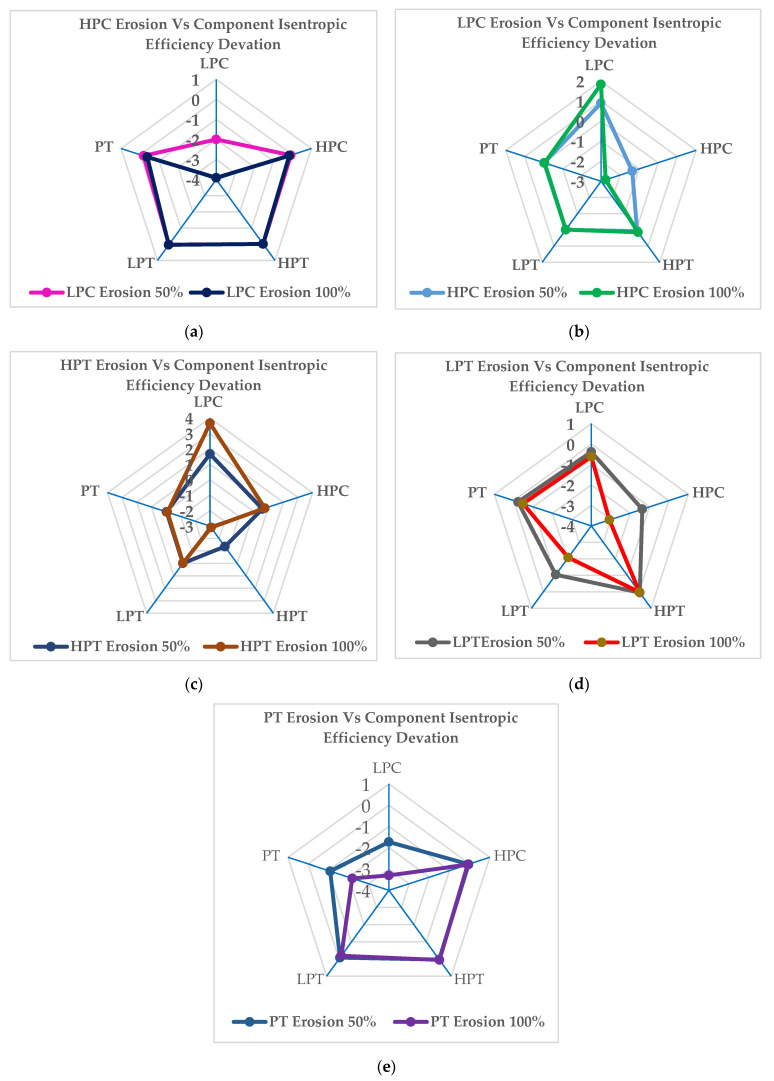
Deviations in the measurement parameters at erosion severity levels of 50% and 100% in the full−load condition for different offending components: (**a**) LPC; (**b**) HPC; (**c**) HPT; (**d**) LPT; (**e**) PT.

Figure 12a illustrates the variation in the component’s isentropic efficiency when the erosion occurs in the low-pressure compressor at the erosion fault severity levels of 50% and 100%. The maximum decreases in isentropic efficiency occur on the compressor itself, by about −2.0% at 50% and −3.09% at the 100% severity level. The maximum deviation in the other component’s isentropic efficiency due to low-pressure compressor erosion is shown maximum for the power turbine, by about −0.18% at 50% and −0.37% at the 100% severity level. Figure 12b depicts the variation in component isentropic efficiency rates when the erosion occurs in the high-pressure compressor at erosion fault severity levels of 50% and 100%. The maximum decreases in isentropic efficiency occur on the compressor itself, by about −1.35% at 50% and about −2.75% at the 100% severity level. The effect on the deviation of the other component’s isentropic efficiency is maximal in the low-pressure compressor, by about 0.9% at 50% and 1.80% at the 100% severity level. Figure 12c depicts the variation in component isentropic efficiency rates when the erosion occurs in the high-pressure turbine at erosion fault severity levels of 50% and 100%. The maximum decreases in isentropic efficiency are shown on the turbine itself, by about 1.67% at 50% and about 3.67% at the 100% severity level. The effect on the deviation of the other component’s isentropic efficiency is maximum in the low-pressure compressor at about −1.35% at 50% and −2.87% at the 100% severity level. Figure 12d illustrates the variation in component isentropic efficiency rates when the erosion occurs in the low-pressure turbine at erosion fault severity levels of 50% and 100%. The decreases in isentropic efficiency occur in the low-pressure compressor, by about −1.03% at 50% and about −2.07% at the 100% severity level. The effect on the deviation of the other component’s isentropic efficiency is maximal in the high-pressure compressor, by about -−1.36% at the 50% severity level and −3.6% at the 100% severity level. Figure 12e illustrates the variation in component isentropic efficiency rates when the fouling occurs in the pressure turbine at erosion fault severity levels of 50% and 100%. The variation in isentropic efficiency rates in the power turbine is about −1.09% at 50% and −2.18% at the 100% severity level. The effect on the deviation of the other component’s isentropic efficiency is maximal in the low-pressure compressor, by about −1.73% at 50% and −3.29% at the 100% severity level.

**Figure 13:**
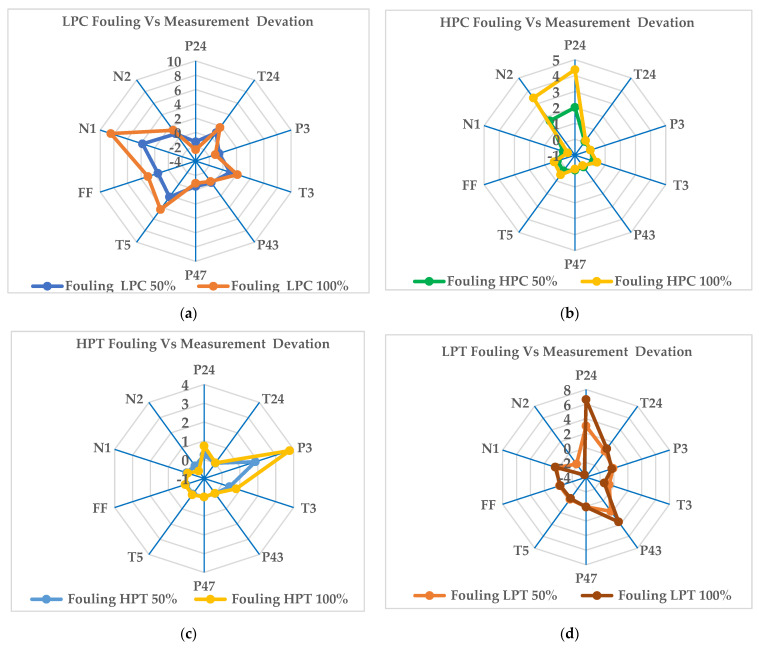

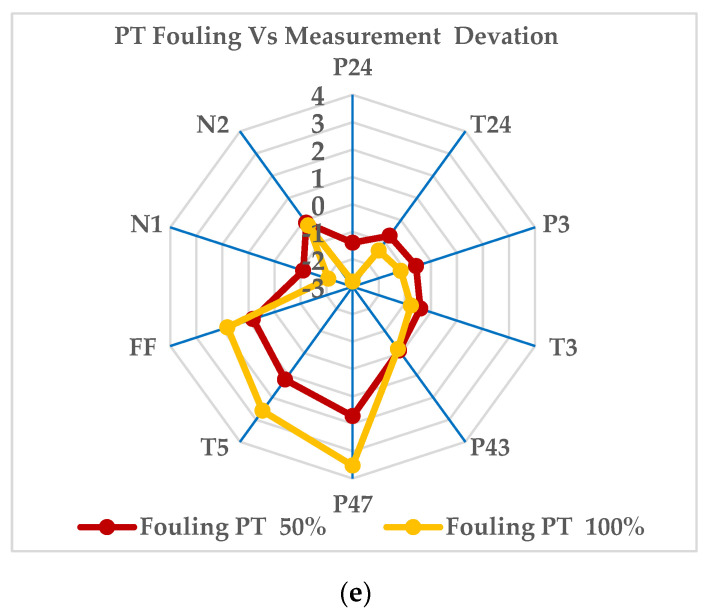
Deviations in the measurement parameters at fouling severity levels of 50% and 100% in the full−load condition for different offending components: (**a**) LPC; (**b**) HPC; (**c**) HPT; (**d**) LPT; (**e**) PT.

There is also a limited number of measurable gas turbine parameters to monitor in a gas turbine. After conducting a correlation analysis, Mohd et al. [34] recommended the ten best three-shaft gas turbine diagnosis set parameters. These are PT4, T24, P3, T3, P43, P47, T5, FF, N1, and N2. Using these parameters, the components of the gas turbine can be diagnosed. In Figure 12 and Figure 13, both fouling and erosion are simulated.

Figure 13a illustrates the variation in measurement parameters when fouling occurs in the low-pressure compressor at fouling fault severity levels of 50% and 100%. The graph shows that the maximum variation occurs for the low-pressure spool speed and low-pressure compressor exit pressure. The low-pressure spool speed increased, by about 3.79% at 50% and about 8.44% at the 100% severity level, and the low-pressure compressor exit pressure drops, as about −1.29% at 50% and about −2.4% at the 100% severity level. In this case, since the low-pressure spool speed and low-pressure compressor exit pressure show the maximum deviation, they can be used for the fault diagnosis model as key parameters. Figure 13b illustrates the variation in measurement parameters when fouling occurs in the high-pressure compressor at the fouling fault severity levels of 50% and 100%. In this simulation, the maximum variation occurs for the low-pressure spool speed and low-pressure compressor exit pressure. The low-pressure spool speed is decreased, by about −0.22% at 50% and about −0.52% at the 100% severity level, and the low-pressure compressor exit pressure is increased, as about 2% at 50% and about 4.3% at the 100% severity level. Since the low-pressure spool speed and low-pressure compressor exit pressure show the maximum deviation, they can be also used for the fault diagnosis model with the quantification level. The variation in measurement parameters when fouling occurs in the high-pressure turbine at the fouling fault severity levels of 50% and 100% is depicted in Figure 13c. This graph shows that the maximum increases occur for the high-pressure compressor exit pressure and high-pressure spool speed. The high-pressure compressor exit pressure increases by about 1.8% at 50% and about 3.77% at the 100% severity level, and the high-pressure spool speed decreases by about −0.17% at 50% and about −0.51% at the 100% severity level. In this case, since the high-pressure compressor exit pressure and high-pressure spool speed show the maximum deviation, they can be used for the fault diagnosis model. Figure 13d illustrates the variation in measurement parameters when the fouling occurs in the low-pressure turbine at fouling fault severity levels of 50% and 100%. Figure 13d shows that the maximum variation occurs for the high-pressure spool speed and low-pressure compressor exit pressure. The high-pressure spool speed decreases by about −1.78% at 50% and about −3.6% at the 100% severity level, while the low-pressure compressor exit pressure increases by about 3.01% at 50% and about 6.62% at the 100% severity level. In this case, since the high-pressure spool speed and low-pressure compressor exit pressure show the maximum deviation, they can be used for the fault diagnosis model. Figure 13e illustrates the variation in measurement parameters when fouling occurs in the power turbine at fouling fault severity levels of 50% and 100%. It shows that the maximum variation occurs for the low-pressure turbine exit pressure and high-pressure compressor exhaust temperature. The low-pressure turbine exit pressure increases by about 1.72% at 50% and about 3.55% at the 100% severity level, and the low-pressure compressor exhaust pressure decreases by about −1.39% at 50% and about −2.81% at the 100% severity level. In this case, the low-pressure turbine exit pressure and high-pressure compressor exhaust temperature can be used for the fault diagnosis model.

**Figure 14:**
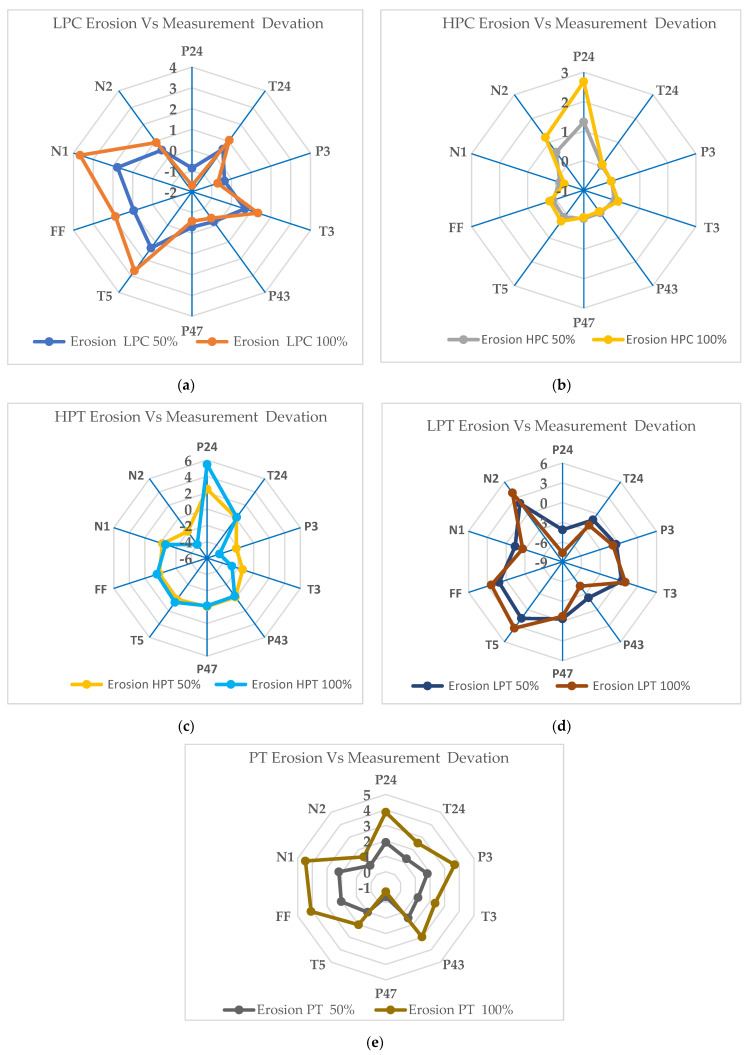
Deviations in measurement parameters at erosion severity levels of 50% and 100% in the full−load condition for different offending components: (**a**) LPC; (**b**) HPC; (**c**) HPT; (**d**) LPT; (**e**) PT.

Figure 14a illustrates the variation in measurement parameters when erosion occurs in the low-pressure compressor at erosion fault severity levels of 50% and 100%. The graph shows that the maximum variation occurs for the low-pressure compressor exit pressure and low-pressure spool speed. The low-pressure compressor exit pressure drops by about −0.86% at 50% and about −1.7% at the 100% severity level, and the low-pressure spool speed increases by about 1.70% at 50% and about 3.66% at the 100% severity level. Based on the simulation results, the erosion in the low-pressure compressor exit pressure and low-pressure spool speed can be used for the fault diagnosis model. Figure 14b illustrates the variation in measurement parameters when the erosion occurs in the high-pressure compressor at erosion fault severity levels of 50% and 100%. The result shows that the maximum variation occurs for the low-pressure compressor exit pressure and low-pressure spool speed. The low-pressure compressor exit pressure increases by about 1.31% at 50% and about 2.68% at the 100% severity level, and the low-pressure spool speed drops by about −0.14% at 50% and about −0.31% at the 100% severity level. Based on the simulation results for the erosion, the low-pressure compressor exit pressure and high-pressure compressor exit pressure can be used for the fault diagnosis model. The variation in measurement parameters when erosion occurs in the high-pressure turbine at fault severity levels of 50% and 100% is depicted in Figure 14c. This graph shows that the maximum increase occurs for the low-pressure compressor exit pressure, with a drop in the high-pressure compressor exit temperature. The low-pressure compressor exit pressure increases by about 2.4% at 50% and about 5.44% at the 100% severity level, and the high-pressure compressor exit temperature drops by about −1.4% at 50% and about −2.7% at the 100% severity level. The minimum deviation occurs in the high-pressure compressor exit pressure, by about −0.065% at 50% and −0.15% at the 100% severity level. In this case, since the low-pressure compressor exit pressure and high-pressure compressor exit temperature show the maximum deviation, they can be used for the fault diagnosis model. Figure 14d illustrates the variation in measurement parameters when erosion occurs in the low-pressure turbine at erosion fault severity levels of 50% and 100%. It shows that the maximum variation occurs for the low-pressure compressor exit and high-pressure spool speed. The low-pressure compressor exit pressure drops by about −4.14% at 50% and about −7.63% at the 100% severity level, while the high-pressure spool speed rises by about 1.97% at 50% and about 3.93% at the 100% severity level. Based on the simulation result, the low-pressure compressor exit and high-pressure spool speed can be used for the fault diagnosis model. Figure 14e illustrates the variation in measurement parameters when erosion occurs in the power turbine at erosion fault severity levels of 50% and 100%. It shows that the maximum variation occurs for the fuel flow rate and low-pressure turbine exit pressure. The fuel flow rate increases by about 2.02% at 50% and about 4.08% at the 100% severity level, and the low-pressure turbine exit pressure decreases by about −0.3% at 50% and about −0.7% at the 100% severity level. To detect erosion in the power turbine, the fuel flow rate and low-pressure turbine exit pressure can be used for the fault diagnosis model.

## 5. Conclusions

In this paper, common gas path faults on the main components of a three-shaft industrial gas turbine in full-load conditions were simulated. The gas path non-linear steady-state model was built using GasTurb 13 commercial software. After the validation of the design point and off-design model, using the relationship between the physical faults and performance parameters and the modifier option for GasTurb 13 component health parameters, the physical faults were implanted to simulate the non-linear steady-state model during deterioration conditions. Then, the influences of fouling and erosion faults on the gas turbine main components were analyzed. The effect of the degraded component on another component was visualized. Among all components, the results proved that the health status of the low-pressure compressor is very crucial. Its deterioration has significant impacts on the other components’ performance. It was also observed that the existence of erosion has a high impact on the downstream components, whereas fouling more affects the upstream components. It was also observed that the upstream components have a greater influence on the downstream components. Furthermore, the simulation result when showed that the variable inlet guide valve had a significant impact on the performance of the gas turbine, especially on the performance of the low-pressure compressor and low-pressure turbine, because the low-pressure turbine is coupled via a common shaft to the low-pressure compressor and the variable inlet guide valve is attached to the low-pressure compressor. The variable inlet guide valve is scheduled with normalized speed. Thus, when the normalized speed decreases due to fouling or erosion, the variable inlet guide valve angle will be changed to change the mass flow rate and to improve the efficiency. This paper will significantly help scientific communities, especially for those who work on gas turbine condition monitoring, because the results of the developed model can be used to gain insights for gas turbine fault detection and diagnostics and can aid in the investigation of the root cause of the performance degradation. An evaluation of the part-load performance when considering the variable inlet guide valve and bleed air remains for future work.

## Figures and Tables

**Table 1:** Design point model input data and technical data.

Parameter	Unit	Value	Source
Power output	MW	26.025	Technical data sheet
Pressure ratio	-	20:1	Technical data sheet
Thermal efficiency	-	35.8	Technical data sheet
Exhaust mass flowrate	Kg/s	92.2	Technical data sheet
Heat rate	KJ/KWh	10,043	Technical data sheet
Turbine inlet temp.	°C	1193	[34]
Exhaust temperature	°C	488	Technical data sheet
LPC rotational speed	RPM	6643	Technical data sheet
HPC rotational speed	RPM	9445	Technical data sheet
FPT rotational speed	RPM	4950	Technical data sheet
LPC stages	-	7	Technical data sheet
HPC stages	-	6	Technical data sheet
HPT stages	-	1	Technical data sheet
LPT stages	-	1	Technical data sheet
FPT stages	-	2	Technical data sheet

**Table 2:** Constraint parameters.

Constraints	Min Value	Optimized Values	Max Value
Thermal efficiency	0.33	0.3585	0.41
Heat rate	8852	10,040.2	10,043.5
Exhaust Temperature (T5)	733	752.076	769.7

**Table 3:** Optimization parameters.

Variables	Min Value	Optimized Value	Max Value
HPT NGV1 Cooling air	0.04	0.061	0.065
HPT Rotor 1 Cooling air	0.03	0.051	0.054
IPT NGV 1 Cooling air	0.008	0.021	0.025
IPT NGV 1 Cooling air	0.008	0.021	0.025
Exhaust pressure ratio	1	1.1620	1.2
IPC Isentropic Efficiency	0.9	0.9	0.95
HPC Isentropic Efficiency	0.9	0.85	0.95
HPT Isentropic Efficiency	0.89	0.8977	0.93
LPT Isentropic Efficiency	0.91	0.9125	0.94
PT Isentropic Efficiency	0.89	0.8963	0.92

**Table 4:** Objective function or figure of merit.

Parameter	Value
Power output (KW)	26,025

**Table 5:** The design point model outputs.

Parameter	Units	Catalogue	GasTurb 13 Model	% Error
Power output	kW	26,025	26,025.5	0.0019
Thermal efficiency	%	35.8	35.8	0
Pressure ratio	-	20:1	20:1	0
Fuel flowrate	kg/s	-	1.53281	-
Lower heating value	MJ/kg	-	47.16	-
Exhaust temperature	K	761	752.5	1.116
Turbine inlet temperature	K	1466	1466	0
Heat rate	kJ/(kWh)	10,043	10,040.2	0.027

**Table 6:** Relationship between the physical faults and health parameters.

Physical Fault	Flow Capacity Change (A)	Isentropic Efficiency Change (B)	Ratio A:B	Range
Compressor fouling	Γ_C_↓	η _C_↓	3:1	(0,−7.5%) (0,−2.5%)
Compressor erosion	Γ_C_↓	η _C_↓	2:1	(0,−4%) (0,−2%)
Turbine fouling	Γ_T_↓	η _T_↓	2:1	(0,−4%) (0,−2%)
Turbine erosion	Γ_T_↓	η _T_↓	2:1	(0,+4%) (0,−2%)

## Data Availability

The data presented in this study are available on request from the corresponding author. The data are not publicly available due to some confidential issues.

## Nomenclature

LPC	Low-pressure compressor
HPC	High-pressure compressor
HPT	High-pressure turbine
LPT	Low-pressure turbine
PT	Power turbine
P24	Low-pressure compressor exit Pressure
T24	Low-pressure compressor exit Temperature
P3	High-pressure compressor exit pressure
T3	High-pressure compressor exit Temperature
P43	High-pressure turbine exit pressure
P47	Low-pressure turbine exit pressure
T5	Power turbine exit temperature
FF	Fuel flow
N1	Low-pressure speed
N2	High-pressure speed
GT	Gas turbine
CC	Combustion chamber
NGV	Nozzle guide vane
VIGV	Variable inlet guide vane

## References

[1] Tahan M., Muhammad M., Karim Z.A.A. (2017). A multi-nets ANN model for real-time performance-based automatic fault diagnosis of industrial gas turbine engines. J. Braz. Soc. Mech. Sci. Eng..

[2] Fentaye A.D., Baheta A.T., Gilani S.I., Kyprianidis K.G. (2019). A Review on Gas Turbine Gas-Path Diagnostics: State-of-the-Art Methods, Challenges and Opportunities. Aerospace.

[3] Li Z., Zhong S., Lin L. (2017). Novel Gas Turbine Fault Diagnosis Method Based on Performance Deviation Model. J. Propuls. Power.

[4] Molla W., Ambri Z., Karim A., Tesfamichael A. (2021). Review on gas turbine condition based diagnosis method. Mater. Today Proc..

[5] Mishra R.K. (2015). Fouling and Corrosion in an Aero Gas Turbine Compressor. J. Fail. Anal. Prev..

[6] Meher-Homji C.B., Chaker M., Bromley A.F. (2009). The fouling of axial flow compressors-Causes, effects, susceptibility and sensitivity. Proc. ASME Turbo Expo.

[7] Morini M., Pinelli M., Spina P.R., Venturini M. (2010). Influence of blade deterioration on compressor and turbine performance. J. Eng. Gas Turbines Power.

[8] Diakunchak I.S. (1991). Performance deterioration in industrial gas turbines. J. Eng. Gas Turbines Power.

[9] Angelos G. (2008). Varelis Technoeconomic Study of Engine Deterioration and Compressor Washing for Military Gas Turbine Engines. Master’s Thesis.

[10] Muthuraman S., Twiddle J., Singh M., Connolly N. (2012). Condition monitoring of SSE gas turbines using artificial neural networks. Insight Non-Destr. Test. Cond. Monit..

[11] Kurz R., Brun K. (2009). Degradation of gas turbine performance in natural gas service. J. Nat. Gas Sci. Eng..

[12] Combined Cycle Performance Deterioration Analysis. https://dspace.lib.cranfield.ac.uk/handle/1826/10462.

[13] Hashmi M.B., Lemma T.A., Karim Z.A.A. (2019). Investigation of the combined effect of variable inlet guide vane drift, fouling, and inlet air cooling on gas turbine performance. Entropy.

[14] Sanaye S., Hosseini S. (2020). Off-design performance improvement of twin-shaft gas turbine by variable geometry turbine and compressor besides fuel control. Proc. Inst. Mech. Eng. Part A J. Power Energy.

[15] Hashmi M.B., Majid M.A.A., Lemma T.A. (2020). Combined effect of inlet air cooling and fouling on performance of variable geometry industrial gas turbines. Alex. Eng. J..

[16] Chen Y.Z., Zhao X.D., Xiang H.C., Tsoutsanis E. (2020). A sequential model-based approach for gas turbine performance diagnostics. Energy.

[17] Merrington G., Kwon O.K., Goodwin G., Carlsson B. (1991). Fault detection and diagnosis in gas turbines. J. Eng. Gas Turbines Power.

[18] Escher P.C. (1995). Pythia: An Object-Orientated Gas Path Analysis Computer Program for General Applications. http://dspace.lib.cranfield.ac.uk/handle/1826/3457.

[19] Saravanamuttoo H.I.H., Maclsaac B.D. (1983). Thermodynamic Models for Pipeline Gas Turbine Diagnostics. http://asme.org/terms.

[20] Kurz R., Brun K. (2001). Degradation in gas turbine systems. J. Eng. Gas Turbines Power.

[21] Saravanamuttoo H.I.H., Zhu P. (1992). Simulation of an Advanced Twin-Spool Industrial Gas Turbine. http://asme.org/terms.

[22] Boyce M.P., Meher-Homji C.B., Lakshminarasimha A.N. (1994). Modeling and Analysis of Gas Turbine Performance Deterioration. http://asme.org/terms.

[23] Macleod J.D., Staff V.T., Laflamme J.C.G. (1992). Implanted Component Faults and Their Effects on Gas Turbine Engine Performance. http://asme.org/terms.

[24] Saravanamuttoo H.I.H., Aker G.F. (1989). Predicting Gas Turbine Performance Degradation Due to Compressor Fouling Using Computer Simulation Techniques. http://asme.org/terms.

[25] Kurz R., Brun K., Wollie M. (2009). Degradation effects on industrial gas turbines. J. Eng. Gas Turbines Power.

[26] Mohammadi E., Montazeri-Gh M. (2014). Simulation of full and part-load performance deterioration of industrial two-shaft gas Turbine. J. Eng. Gas Turbines Power.

[27] Qingcai Y., Li S., Cao Y., Zhao N. (2016). Full and Part-Load Performance Deterioration Analysis of Industrial Three-Shaft Gas Turbine Based on Genetic Algorithm. http://proceedings.asmedigitalcollection.asme.org/pdfaccess.ashx?url=/data/conferences/asmep/89511/.

[28] Salilew W.M., Karim Z.A.A., Lemma T.A. (2022). Investigation of fault detection and isolation accuracy of different Machine learning techniques with different data processing methods for gas turbine. Alex. Eng. J..

[29] Razak A.M.Y. (2013). Gas Turbine Performance Modelling, Analysis and Optimisation.

[30] Razak A.M.Y. (2007). Industrial Gas Turbines: Performance and Operability.

[31] Kurzke J. 95-GT-147 Advanced User-Friendly Gas Turbine Performance Calculations on A Personal Computer. http://asmedigitalcollection.asme.org/GT/proceedings-pdf/GT1995/78828/V005T16A003/2406887/v005t16a003-95-gt-147.pdf.

[32] Turbine G., Combustion I. (2020). 13.1 Single-Shaft Gas Turbine Engine. Design-Point Calculations of Industrial Gas Turbines.

[33] Ao S.I., Gelman L., Hukins D.W.L., Hunter A., Korsunsky A. (2018). International Association of Engineers. Design and Off-Design Operation and Performance Analysis of a Gas Turbine.

[34] Jasmani M.S., Li Y.G., Ariffin Z. (2011). Measurement selections for multicomponent gas path diagnostics using analytical approach and measurement subset concept. J. Eng. Gas Turbines Power.

[35] Gao J.H., Huang Y.Y. Modeling and simulation of a aero turbojet engine with GasTurb. Proceedings of the 2011 International Conference on Intelligence Science and Information Engineering, ISIE 2011.

[36] Kurzke J. (2007). About Simplifications in Gas Turbine Performance Calculations. www.gasturb.de.

[37] (2008). EFSTRATIOS NTANTIS. Capability Expansion of Non Linear Gas Path Analysis. Ph.D. Thesis.

